# Effects of plant functional group removal on CO_2_ fluxes and belowground C stocks across contrasting ecosystems

**DOI:** 10.1002/ecy.3170

**Published:** 2020-10-06

**Authors:** Roger Grau‐Andrés, David A. Wardle, Michael J. Gundale, Claire N. Foster, Paul Kardol

**Affiliations:** ^1^ Department of Forest Ecology and Management Swedish University of Agricultural Sciences (SLU) Umeå Sweden; ^2^ Asian School of the Environment Nanyang Technological University Singapore Singapore; ^3^ Fenner School of Environment and Society The Australian National University Canberra Australian Capital Territory Australia

**Keywords:** biodiversity, boreal forest, context‐dependency, moss, net ecosystem exchange, plant–soil interaction, primary productivity, respiration, shrub, soil carbon, soil microclimate, understory

## Abstract

Changes in plant communities can have large effects on ecosystem carbon (C) dynamics and long‐term C stocks. However, how these effects are mediated by environmental context or vary among ecosystems is not well understood. To study this, we used a long‐term plant removal experiment set up across 30 forested lake islands in northern Sweden that collectively represent a strong gradient of soil fertility and ecosystem productivity. We measured forest floor CO_2_ exchange and aboveground and belowground C stocks for a 22‐yr experiment involving factorial removal of the two dominant functional groups of the boreal forest understory, namely ericaceous dwarf shrubs and feather mosses, on each of the 30 islands. We found that long‐term shrub and moss removal increased forest floor net CO_2_ loss and decreased belowground C stocks consistently across the islands irrespective of their productivity or soil fertility. However, we did see context‐dependent responses of respiration to shrub removals because removals only increased respiration on islands of intermediate productivity. Both CO_2_ exchange and C stocks responded more strongly to shrub removal than to moss removal. Shrub removal reduced gross primary productivity of the forest floor consistently across the island gradient, but it had no effect on respiration, which suggests that loss of belowground C caused by the removals was driven by reduced litter inputs. Across the island gradient, shrub removal consistently depleted C stocks in the soil organic horizon by 0.8 kg C/m^2^. Our results show that the effect of plant functional group diversity on C dynamics can be relatively consistent across contrasting ecosystems that vary greatly in productivity and soil fertility. These findings underline the key role of understory vegetation in forest C cycling, and suggest that global change leading to changes in the relative abundance of both shrubs and mosses could impact on the capacity of boreal forests to store C.

## Introduction

Terrestrial ecosystems are threatened by environmental changes, including warmer climate, altered precipitation regimes, increased nitrogen deposition, and changes in land management (Gauthier et al. 2015). Such environmental changes often result in large shifts in plant community composition and loss of species and functional groups, and concomitant changes in ecosystem processes, including the cycling of carbon (C). Changes in dominant plant species and their trait spectra can greatly impact ecosystem C gain through differences in gross primary productivity and carbon use efficiency, as well as C loss through differences in aboveground and rhizosphere respiration (Högberg and Read 2006, De Deyn et al. 2008). Further, plant community composition controls rates of C loss by heterotrophic respiration through influencing the chemical composition and decomposability of aboveground and belowground litter inputs (Metcalfe et al. 2011), the composition of root‐associated biota (Clemmensen et al. 2015), and soil microbial activity through changing the belowground thermal and moisture regime (Sun et al. 2017). As such, by influencing ecosystem C exchange, plant community composition is a dominant driver of the long‐term accumulation of C belowground (Jonsson and Wardle 2010, Clemmensen et al. 2013).

While the effects of shifts in plant community composition and loss of diversity on C cycling have received growing attention (Chen et al. 2016, Li et al. 2018, Smith et al. 2020), how these effects vary across ecosystems is not well understood. Removal experiments, where the same plant species or functional groups are removed across different environmental settings, have shown that the effect of removals on drivers of C cycling (i.e., soil respiration, litter decomposition rates, net primary productivity) vary along abiotic and biotic gradients, including gradients in ecosystem productivity (Wardle and Zackrisson 2005, Fanin et al. 2018) and nutrient availability (Suding et al. 2008, McLaren and Turkington 2011). However, experiments testing the context dependency of the loss of plant species or functional groups are still scarce, and we are not aware of any experimental studies that have explored how removals of the same species or functional groups in contrasting ecosystems affects the C balance or long‐term C sequestration. A better understanding of the effects of plant diversity loss on C cycling and C sequestration, and how these effects are mediated by environmental context, is however important for predicting how global change may impact the capacity of ecosystems to store C.

Boreal forests store globally substantial amounts of C (~32% of global forest C with 60% of this in soils; Pan et al. 2011). Although trees account for most of the plant biomass in boreal forests, the understory vegetation, which is often dominated by shrubs and mosses, is an important driver of ecosystem C dynamics (Kolari et al. 2006, Turetsky et al. 2012). For example, ericaceous dwarf shrubs and feather mosses, which are ubiquitous across large parts of the boreal biome, contribute substantially to net primary productivity due to their high turnover rates (Wardle et al. 2012). Further, shrubs and mosses can influence rates of soil microbial decomposition through variation in their litter quality (Lang et al. 2009) and by regulating soil temperature and moisture (De Long et al. 2016, Sun et al. 2017). However, we do not know how the effects of shrubs and mosses on boreal forest C dynamics are mediated by environmental context, such as changes associated with boreal forest succession. Early‐succession boreal forests tend to be dominated by tree and understory vegetation with high requirements for light and nutrients (e.g., *Pinus* spp., *Betula* spp., *Rubus idaeus*, *Vaccinium myrtillus*) relative to species that dominate late‐succession forests (e.g., *Abies* spp., *Picea* spp, *Sphagnum* spp., *Empetrum hermaphroditum*; Wardle et al. 1997, Hart and Chen 2006, Bergeron and Fenton 2012). This has important implications for the functioning of the forest floor: for example, rates of understory net primary productivity and litter decomposition are higher in early‐successional, productive forests dominated by resource‐acquisitive shrubs than in later‐succession forests dominated by resource‐conservative species (Nilsson and Wardle 2005). Moreover, lower decomposition rates in late‐succession forests leads to greater soil organic matter accumulation than in early‐successional forests (Bergeron and Fenton 2012, Clemmensen et al. 2015). Therefore, understanding how boreal forest C cycling responds to changes in understory vegetation across a wide range of environmental conditions is important for predicting how global change may affect C stocks (Gauthier et al. 2015).

Here, we investigated the effects of loss of understory plant functional groups on forest floor CO_2_ exchange and C stocks in boreal forests, and whether these effects varied among contrasting independent ecosystems or depended on environmental context. For this, we used the longest‐running plant biodiversity manipulation experiment across contrasting ecosystems in existence, which was established in 1996 (Wardle and Zackrisson 2005) and is still ongoing. The experiment is set up on each of 30 forested islands in northern Sweden that represent highly contrasting ecosystems. For larger islands, where lightning‐ignited wildfires have occurred more frequently, forests are early‐successional and are dominated by resource‐acquisitive species (e.g., *V. myrtillus*), and have high ecosystem productivity and high soil fertility. Conversely, forests in smaller islands are late‐successional and are dominated by resource‐conservative species (e.g., *E. hermaphroditum*), and have low ecosystem productivity and low soil fertility (Wardle et al. 2003, 1997). The part of the experiment used in the present study includes factorial removal of dwarf shrubs and feather mosses on each island, allowing us to test the effect of 22 yr of removal of these two plant functional groups, singly and in combination, on C cycling and storage across a strong boreal forest productivity gradient. We tested the following hypotheses: (1a) Shrub and moss removal will reduce forest floor ecosystem respiration (ER) and gross primary productivity (GPP). Greater decreases in GPP than in ER with removals will result in increases in net ecosystem exchange (NEE), i.e., weaken the forest floor CO_2_ sink. (1b) The effects of shrub removal on increasing NEE will be greatest on large and medium islands because they are dominated by more productive, resource‐acquisitive species compared to the resource‐conservative species that dominate small islands (Wardle et al. 2012). Meanwhile the effects of moss removal on increasing NEE will be greatest on small islands because they have the most moss biomass (Lagerström et al. 2007); (2a) A weaker CO_2_ sink resulting from shrub and moss removal will result in a long‐term reduction in belowground C stocks. (2b) Further, in alignment with our first hypothesis, shrub removal will have the greatest effect on reducing C stocks on large islands, while the effect of moss removal will be greatest on small islands. By testing these hypotheses in combination we aimed to understand the long‐term effects of changes in understory composition on the capacity of boreal forests to store C, and how these effects vary across ecosystems.

## Materials and Methods

### Study site and experimental design

The study system is a set of 30 boreal forested lake islands in northern Sweden (lakes Hornavan and Uddjaure, 65°56.7′ N to 66°09.6′ N, 17°42.9′ E to 17°52.3′ E; Appendix S1: Fig. S1) that have been extensively studied (Wardle et al. 2012, 1997, Fanin et al. 2018). The mean annual precipitation is 750 mm, and the mean temperature is +13°C in July and −14°C in January. The region of our study is not underlain by permafrost (Gisnås et al. 2017). All islands were formed following the retreat of land ice about 9000 yr ago. The only major extrinsic factor that varies among islands is the history of lightning ignited wildfire, with larger islands having burned more frequently with stand‐replacing fires than have smaller islands because of their larger area to intercept lightning (Wardle et al. 2003, 1997). Consistent with previous work from this study system (Wardle et al. 2003, 2012, Fanin et al. 2018, Kardol et al. 2018, Fanin et al. 2019) we classified our 30 islands into three size classes with 10 islands in each class: large (>1.0 ha, mean time since fire 585 yr), medium (0.1–1.0 ha, mean time since fire 2,180 yr) and small (<0.1 ha, mean time since fire 3,250 yr). In the prolonged absence of wildfires the islands undergo ecosystem retrogression as a result of vegetation succession from dominance of species that are resource acquisitive (e.g., *Pinus sylvestris*, *Vaccinium myrtillus*) to dominance of more conservative species (e.g., *Picea abies*, *Empetrum hermaphroditum*). This is associated with declining soil nutrient availability and reduced plant biomass and productivity (Wardle et al. 2003, Clemmensen et al. 2013). For example, from large and medium islands to small islands, soil mineral nitrogen declines from on average 48.2–25.3 μg N/g, soil mineral phosphorus from 40.7 to 24.4 μg P/g, and tree biomass density from 8.5 to 3.2 kg/m^2^. Moreover, vegetation on smaller islands with a longer time since fire produce more recalcitrant litter and fungal necromass, leading to slower decomposition and nutrient mineralization and to higher soil C sequestration (Jonsson and Wardle 2010, Clemmensen et al. 2015). Further details on the study system are presented in Appendix S1: Table S1.

**Fig. 1:**
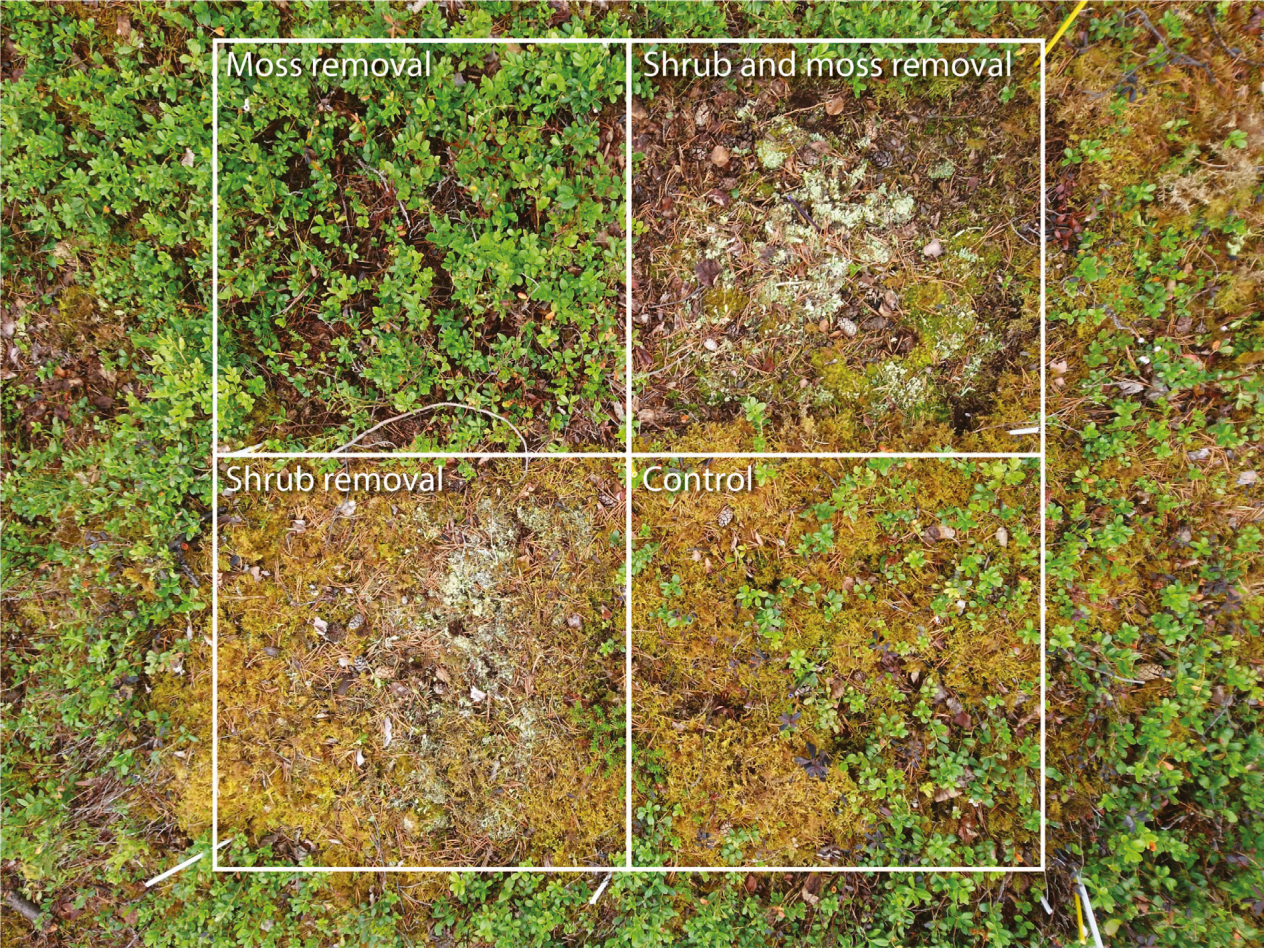
Example of plant functional group removal plots in a medium‐size island. The shrub community is dominated by *Vaccinium vitis‐idaea*, with *Vaccinium myrtillus* and *Empetrum hermaphroditum* also present. *Hylocomium splendens* dominates the moss layer. [Color figure can be viewed at wileyonlinelibrary.com]

To study the effects of plant functional group loss on ground‐level CO_2_ fluxes and ecosystem C stocks, we used an ongoing biodiversity manipulation experiment established in 1996 (Wardle and Zackrisson 2005). From this experiment, we used four plots on each of the 30 islands, which represent the four treatment combinations of no removals, removal of feather mosses only, removal of dwarf shrubs only, and removal of both feather mosses and dwarf shrubs (Fig. 1). Shrubs and mosses represent the two functional groups of plants that comprise most (~97%) of the understory biomass on these islands (Wardle et al. 2003, Jonsson et al. 2015). Removals have been performed on each plot at the peak of the growing season every year since 1996 and are ongoing. All plots are 55 × 55 cm but we only used the innermost 45 × 45 cm for sampling. Further details of this experiment are given in Wardle and Zackrisson (2005), Fanin et al. (2018) and Fanin et al. (2019).

### Forest floor CO_2_ flux

We estimated forest floor CO_2_ fluxes in each plot (*N* = 120) between 30 July to 16 August 2018, 22 yr after the removal experiment started, using the approach described by Wardle et al. (2016). To do this, we measured the CO_2_ concentration in a 40 × 40 × 40 cm clear acrylic chamber (92% light transmittance) over ~4 minutes at 15 s intervals using a CO_2_ analyzer (Vaisala GMP343, Helsinki, Finland). A 6 cm diameter fan (4000 RPM, air flow = 0.49 m^3^/min) mixed the air in the chamber during measurements. Photosynthetically active radiation (PAR) at the top of the chamber was measured simultaneously with CO_2_ measurements using a PAR meter (Apogee Instruments MQ‐500, Logan, Utah, USA), and the temperature of the top 12 cm of soil was measured immediately after CO_2_ measurements using a Sunartis E514 thermometer. To seal the chamber to the ground, flexible plastic sheets attached to the outer side of the panels were pressed to the ground with chains and rocks. We ventilated the chambers for ~90 s between measurements. To estimate moss moisture content, we collected ten representative moss specimens immediately after flux measurements from each plot for which mosses had not been removed. The samples were kept in air‐tight containers and weighed on the same day, and moisture content was calculated gravimetrically after drying the samples.

Fluxes of CO_2_ were calculated as the initial rate of CO_2_ change by regression analysis, following Pedersen et al. (2010) as implemented in the package HMR (Pedersen 2017) in R version 3.6.1 (R Core Team 2019). Net ecosystem exchange (NEE) was estimated with the sealed chamber either uncovered or covered with mesh of varying light transmittance, and ecosystem respiration (ER) was calculated from a single measurement with the chamber covered in an opaque plastic sheet (0% light transmittance). We note that forest floor respiration include the contribution of tree roots, in addition to respiration from soil biota and non‐removed understory vegetation (Wardle et al. 2016). Gross primary productivity (GPP) was calculated as ER minus NEE. To account for differences in light conditions between measurement times and locations, we standardized NEE and GPP to a common PAR by taking approximately five measurements per plot (*N* = 583), each covering the chamber with mesh of varying light transmittance: 92% (no cover), 81%, 72%, 55%, and 35%. An exponential three‐parameter decay model was fitted to the relationship between NEE and PAR in each plot (Metcalfe et al. 2013) using the functions nls and SSasymp in R. When a nonlinear fit was not justified (i.e., where shrub cover or PAR range were low), a linear model was fitted instead. The model was then used to predict NEE for a target PAR. Based on the overall range of maximum PAR, each island was assigned to one of three groups (low, medium or high PAR at the time of measurement), with median PAR of 70, 250 and 650 μmol·m^−2^·s^−1^, respectively. We chose standardizing to three PAR levels, rather than a single level, to avoid extrapolating beyond the observed PAR range in each island while still standardizing to a representative PAR of the observed conditions (mean = 505 μmol·m^−2^·s^−1^, range = 48–1137 μmol·m^−2^·s^−1^; Appendix S1: Table S2). To keep a balanced design, the same number of islands was assigned to each PAR level in each of the three island size classes (large, medium, and small). Within each size class, NEE was standardized to PAR = 70 μmol·m^−2^·s^−1^ in one island, 250 μmol·m^−2^·s^−1^ in five islands and 650 μmol·m^−2^·s^−1^ in four islands.

### Aboveground and belowground carbon stocks

Shrub biomass was estimated in each plot (*N* = 120) from 30 July to 16 August 2018. To do this, we used the point intercept method as described in Wardle et al. (2003) to record the number of times that 100 downward‐projecting rods intercepted each of the three dominant shrub species (*V. myrtillus*, *Vaccinium vitis‐idaea*, and *E. hermaphroditum*), which collectively represent 98% of the total biomass in the shrub layer on the islands. For each species, we then converted the number of intercepts to biomass using allometric equations developed for that species by Wardle et al. (2003). Similarly, we estimated the biomass in each plot of the two most common feather moss species (*Hylocomium splendens* and *Pleurozium schreberi*, which account for 96% of moss cover) by measuring the thickness of the living moss in 10 equally spaced positions in the plot and then converting to biomass following allometric equations presented by Lagerström et al. (2007). We converted biomass to C using a factor of 0.5 for shrubs (Pasalodos‐Tato et al. 2015, Steinwandter et al. 2019) and 0.4 for mosses (Delgado et al. 2013, Maksimova et al. 2013).

To estimate soil organic C content, we took a single core of the entire soil organic horizon (i.e., the “humus” layer) in each plot between 12–17 August 2018. We sampled soil organic horizons <30 cm thick using a stainless steel cylinder (internal diameter 3 cm; Fanin et al. 2018) and thicker horizons (maximum 91.5 cm) using a PN425 JMC Sub‐Soil Probe PLUS (JMC Soil Samplers, Newton, Iowa, USA; internal diameter 3 cm; Clemmensen et al. 2013). To account for potential differences in organic matter content with depth, each core was divided into one to four subsamples (mean 2.5) based on apparent humification (Soil Survey Staff 2015). The 294 resulting subsamples were each oven dried at 60°C and sieved through a 2mm sieve, after removing bigger roots, and then ashed at 550°C for 6 h to calculate the percentage of organic matter. We used a conversion factor of 0.511 g C/g organic matter to calculate C content, based on previous analyses of the top 10 cm of the organic soil horizon in our study system (Wardle et al. 2020). The conversion factor did not differ between removal treatments or island size classes (*P* > 0.4; Wardle et al. 2020). Within each plot, we summed the C content in the subsamples to obtain total soil organic C. Additionally, to account for within‐plot heterogeneity in the thickness of the soil organic horizon, we took four non‐destructive measurements per plot over 15–19 July 2019 using a 1.1 m long, 5 mm diameter, metal rod. To incorporate this within‐plot variability into the C content estimates from the cores, we first modeled the C content in each core from the soil organic horizon thickness as measured by the hole left after core removal in the field (marginal *R* ^2^ = 0.76, *N* = 120; Appendix S1: Fig. S2). This model also included island size class to account for differences in bulk density between island size classes (*F*_2,27_ = 5.5, *P* = 0.01). Removal treatment was not included because it did not affect bulk density overall (*F*_3,81_ = 1.1, *P* = 0.4) or within‐island size classes (interaction *F*_6,81_ = 1.3, *P* = 0.3), and led to a poorer‐fitting model (ΔAIC = +15). We then used the modeled relationship between thickness of the soil organic horizon and C content to predict soil organic C content for each plot using the thickness measurements for that plot provided by the non‐destructive probing.

### Soil microclimate

To examine whether alteration of the soil thermal regime due to plant functional group loss could be an important driver of soil C dynamics, on each island, we monitored surface soil temperatures in plots where both shrubs and mosses had been removed and in control (non‐removal) plots. We used temperature loggers (iButtons; Maxim Integrated, San Jose, CA, USA) buried 2 cm below the soil surface (i.e., below the moss and litter layer when present) to record soil temperature every 255 min from 15 August 2018 to 14 July 2019. We calculated mean daily soil temperature and daily temperature range in each plot. For each island we determined the change in soil temperature regime due to the removal of shrubs and mosses by subtracting mean daily temperature (and, separately, daily temperature range) in plots where shrubs and mosses had been removed from mean daily temperature (and range) in control plots. Mean daily temperature and range were then averaged for each island and season. We note that data from both 2018 and 2019 contributed to the summer average. Logger malfunctions meant temperature records were available for only 27 of the 30 islands.

We measured the moisture content of the top 12 cm of the soil organic horizon in each plot using a soil moisture probe (Hydrosense II, Campbell Scientific, Logan, Utah, USA), taking four measurements per plot. We monitored soil moisture in July 2018 and again in August 2018 (during CO_2_ flux monitoring). For each plot, we averaged all soil moisture content measurements for both July and August to obtain a single value for each plot.

### Statistical analyses

We used R version 3.6.1 for all data analysis and plotting. To account for the nested structure of the experimental design (because removal treatments were nested within islands), we used linear mixed effects models (package nlme; Pinheiro et al. 2018) that included island identity as a random effect. We tested the effect of shrub removal, moss removal and island size on CO_2_ fluxes (separately for NEE, ER, and GPP), soil organic C stocks and soil moisture content by fitting the interactions among these factors as fixed effects. To account for potential differences in root respiration among islands, we initially included island‐level tree biomass density (Wardle et al. 2003) in the ER model, but this variable had no effect on ER (*F*_1,26_ = 0.1, *P* = 0.8) and led to a poorer‐fitting model (ΔAIC = +2), and therefore was not included in the final models. Shrub C content was analyzed with the interaction between moss removal and island size as a fixed effect, and moss C with the interaction between shrub removal and island size as a fixed effect. To analyze changes in mean daily soil temperature and range due to plant functional group removal we used the interaction between size class and season (i.e., spring, March–May; summer, June–August; autumn, September–November; winter, December–February) as a fixed effect. Island identity was included as a random effect to account for temporal correlation.

We checked model assumptions by plotting residuals against fitted values, and against levels of fixed factors, and used a constant variance function (varIdent) to account for variance heterogeneity among removal plots and/or island size classes when appropriate (Zuur et al. 2009). A log transformation was also necessary to stabilize the variance when analyzing moss C stocks and for predicting soil organic C from thickness of the soil organic horizon. In interaction models, multiple comparisons within the levels of each factor were calculated using the function glht in multcomp (Hothorn et al. 2008). Marginal *R* ^2^, i.e., the proportion of the total variance explained by the fixed effects, and conditional *R* ^2^, i.e., the variance explained by both fixed and random effects (Nakagawa et al. 2017), were calculated using the function r.squaredGLMM in MuMIn (Barton 2019).

## Results

### Forest floor CO_2_ flux

**Fig. 2:**
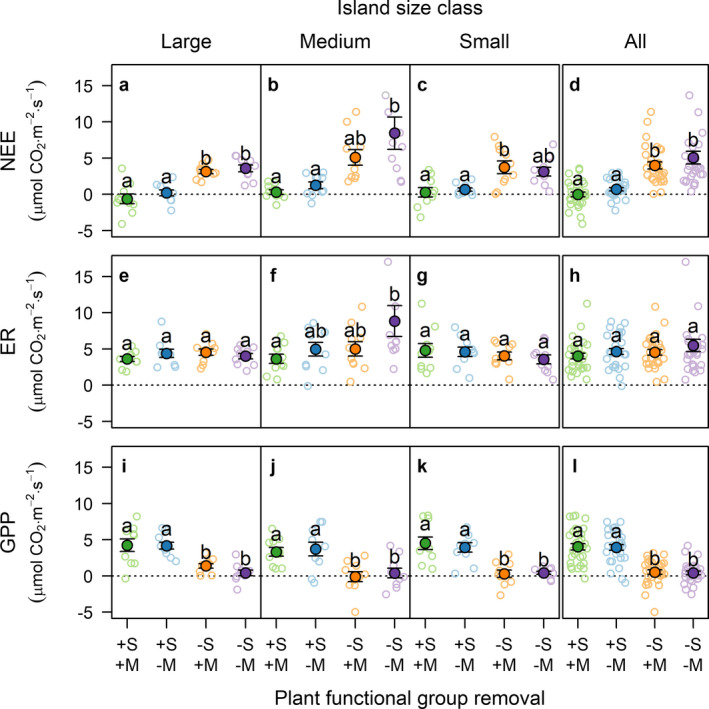
Forest floor CO_2_ fluxes, including (a–d) net ecosystem exchange (NEE; positive values indicate CO_2_ release), (e–h) ecosystem respiration (ER), and (i–l) gross primary productivity (GPP) in response to plant functional group removal and island size class. +S+M, non‐removal plots (controls); +S‐M, moss removal; −S+M, shrub removal; −S‐M, shrub and moss removal. Solid circles are mean ± SE of individual islands (open circles; *N* = 10). Within panels, same lowercase letters indicate differences between removal treatments are not statistically significant (post‐hoc comparisons based on Tukey's test, α = 0.05). Overall differences between island size classes were not significant. To aid visualization, two extreme observations were not plotted: ER = 25.8 and NEE = 24.1 μmol CO_2_·m^−2^·s^−1^, both from the same ‐S‐M plot in a medium island. Test statistics for the models underpinning this figure are given in Appendix S1: Table S3. [Color figure can be viewed at wileyonlinelibrary.com]

Forest floor CO_2_ fluxes were impacted by plant functional group removals (Fig. 2; Appendix S1: Table S3). Overall, net ecosystem exchange (NEE) increased by 4.2 ± 0.5 μmol CO_2_·m^−2^·s^−1^ (mean ± SE) due to shrub removal and by 0.9 ± 0.7 μmol CO_2_·m^−2^·s^‐1^ due to moss removal (Fig. 2d); these effects were consistent across all island size classes (Fig. 2a–c). There was also some evidence (*F*_2,81_ = 2.4, *P* = 0.09) of an interactive effect of shrub removal and island size on NEE through removal increasing NEE more on medium islands compared to large and to small islands. Shrub and moss removals had no significant main effects on ecosystem respiration (ER; Fig. 2h), but there was an interactive effect of shrub removal and island size (Appendix S1: Table S3), through shrub removal substantially increasing ER on medium islands, but not on large and small islands (Fig. 2e–g). Meanwhile gross primary productivity (GPP) was reduced overall by shrub removal (i.e., lowered CO_2_ uptake) by on average 3.5 ± 0.4 μmol CO_2_·m^−2^·s^‐1^ while moss removal had no effect (Fig. 2l; Appendix S1: Table S3). The effect of shrub removal on GPP was consistent across all island size classes (Fig. 2i–k).

### Aboveground and belowground C stocks

**Fig. 3:**
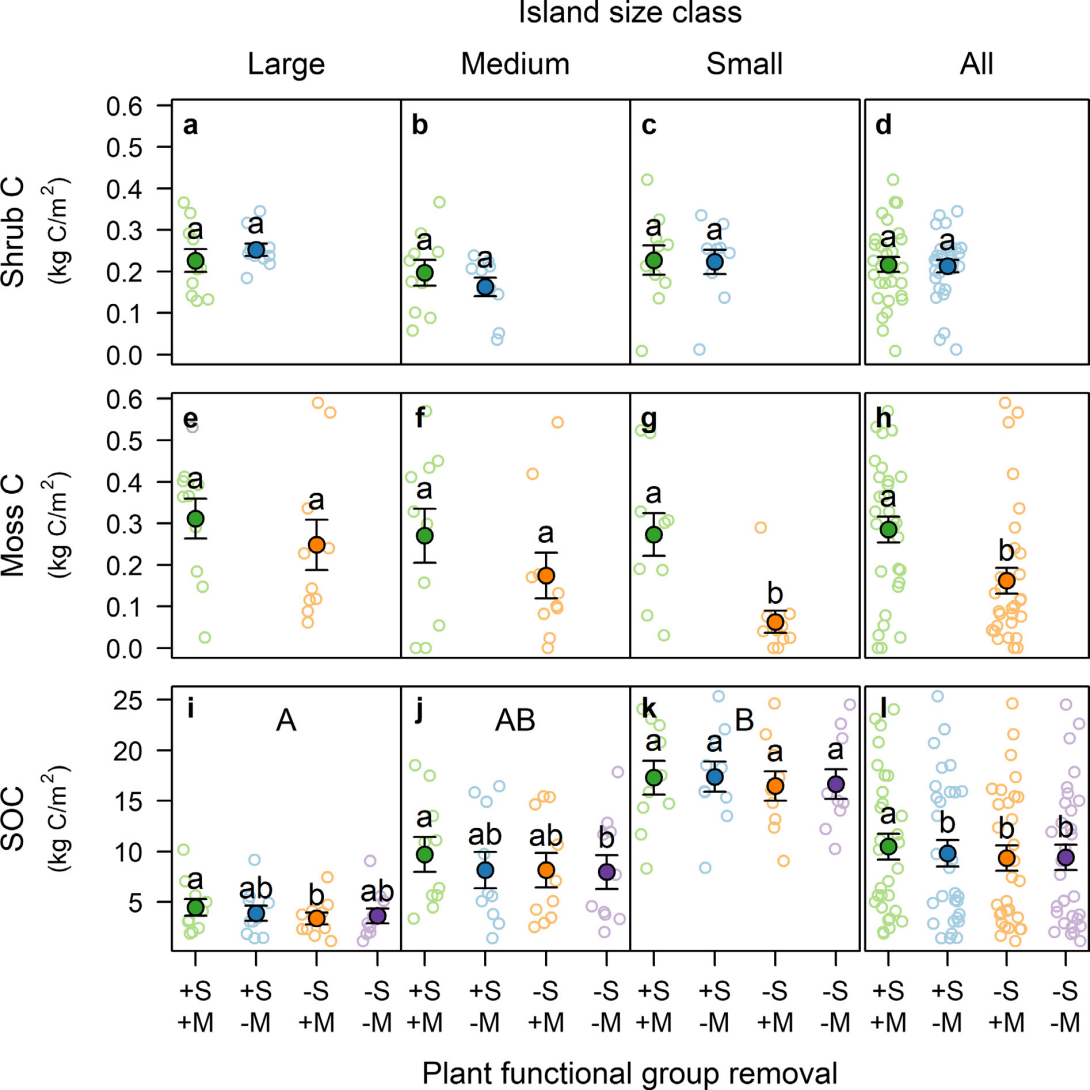
Carbon stocks in the aboveground component of (a–d) shrubs, (e–h) mosses, and (i–l) in the soil organic horizon (i.e., soil organic carbon, SOC), in response to plant functional group removal and island size class. +S+M, non‐removal plots (controls); +S‐M, moss removal; −S+M, shrub removal; −S‐M; shrub and moss removal. Solid circles are mean ± SE of individual islands (open circles; *N* = 10). Within panels, same lowercase letters indicate differences between removal treatments are not statistically significant (post‐hoc comparisons based on Tukey's test, α = 0.05). Within metrics, same uppercase letters indicate overall differences between island size classes are not significant, and no uppercase letters indicate that all pairwise differences are not significant. Test statistics for the models underpinning this figure are given in Appendix S1: Table S4. [Color figure can be viewed at wileyonlinelibrary.com]

Carbon stocks of the aboveground shrub biomass were not affected by moss removal (Fig. 3a–d), but C stocks of the aboveground shrub biomass were lowest in medium islands (Appendix S1: Table S4). In contrast, shrub removal decreased moss C stocks on average by 0.12 ± 0.04 kg C/m^2^, and this decrease was stronger on small islands than on medium and larger islands (Fig. 3e–h; Appendix S1: Table S4). Non‐removal plots were dominated by *V. myrtillus* (196 ± 30 g biomass/m^2^) and *V. vitis‐idaea* (166 ± 23 g/m^2^) on large islands, by *V. vitis‐idaea* (327 ± 64 g/m^2^) on medium islands, and by *V. vitis‐idaea* (201 ± 38 g/m^2^) and *E. hermaphroditum* (192 ± 63 g/m^2^) on small islands. In non‐removal plots, 95% of feather moss biomass was from *H. splendens* (410 ± 60 kg/m^2^ on average) and from *P. schreberi* (310 ± 70 kg/m^2^). The biomass of each species did not change across the island size gradient (*H. splendens F*_2,27_ = 0.5, *P* = 0.6; *P. schreberi F*_2,27_ = 0.1, *P* = 0.9). Shrub removal decreased soil organic C stocks on average by 0.8 ± 0.2 kg C/m^2^, while moss removal had no effect (*F*_1,81_ = 2.4, *P* = 0.12). However, there was an interactive effect of shrub and moss removals whereby when both shrubs and mosses were removed soil organic C decreased less than expected based on their individual effects (Fig. 3l; Appendix S1: Table S4). Soil organic C stocks decreased with increasing island size (Fig. 3i–k), but no interaction between island size and plant functional group removal occurred (Appendix S1: Table S4).

### Soil microclimate

**Fig. 4:**
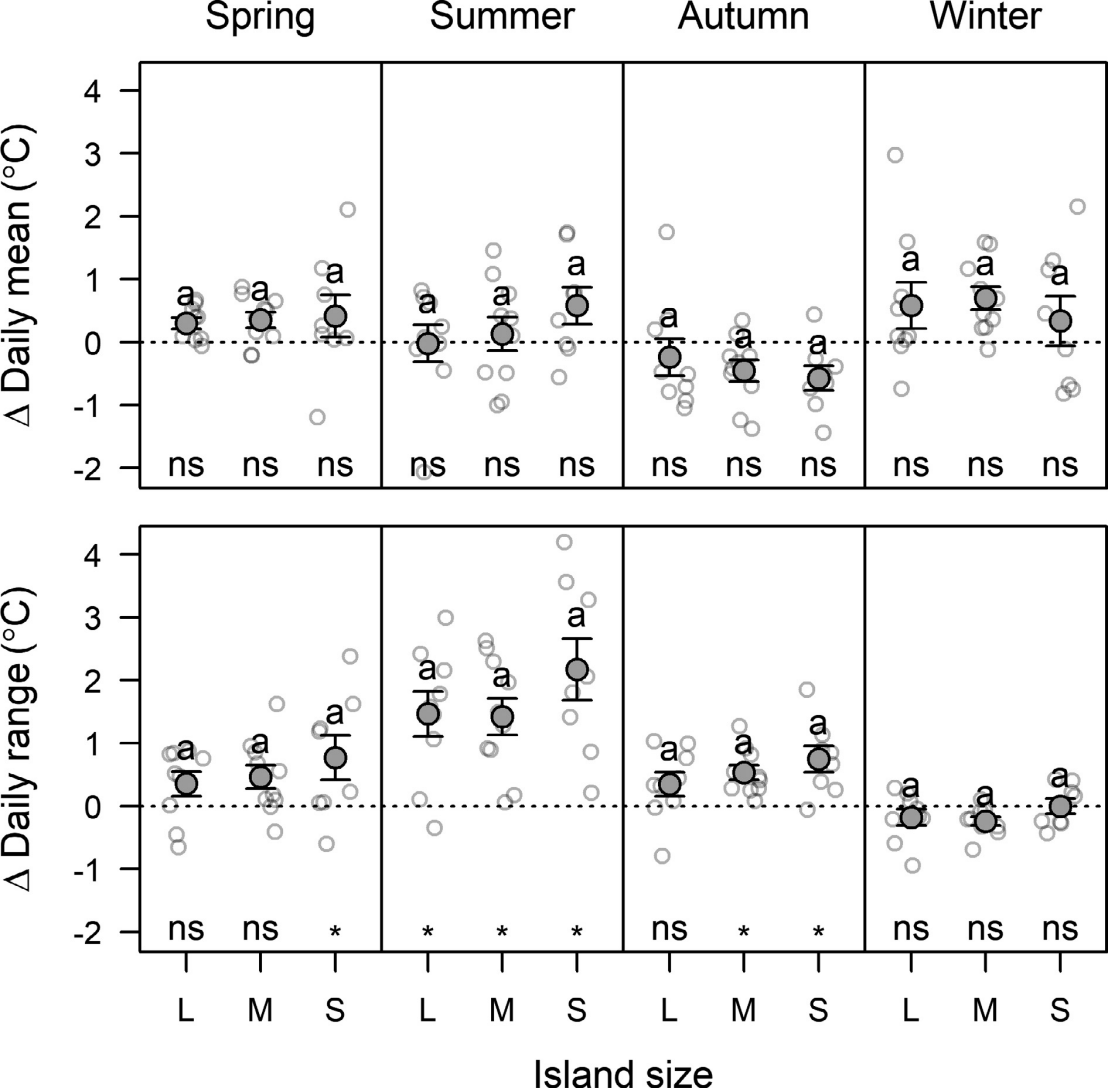
Difference in mean soil daily temperature and in daily temperature range due to the combined removal of shrubs and mosses for large (L), medium (M), and small (S) islands, calculated as daily mean (and range) in removal plots minus in control plots, i.e., positive values indicate removal plots are warmer (ΔMean) or have greater diurnal variation (ΔRange) than control plots. Open circles are island‐level averages (*N* = 10); solid circles are mean ± SE. Within panels, same letters indicate that differences between island size classes are not statistically significant, and * indicate values that are significantly different from zero (post‐hoc comparisons based on Tukey's test, α = 0.05; ns, is not significant). Test statistics for the models underpinning this figure are provided in Appendix S1: Table S5.

Soil thermal regime changed in response to the combined removal of shrubs and mosses. Across all island size classes, the removals led to warmer soil over the entire measuring period (+0.2° ± 0.1°C), and in spring (+0.4° ± 0.1°C) and winter (+0.6° ± 0.2°C), but to cooler soil in autumn (−0.4° ± 0.1°C; Fig. 4). Within each season, the change in mean soil daily temperature caused by the removals was consistent across the island size gradient (Appendix S1: Table S5). Removal of shrubs and mosses increased daily temperature range by on average 0.6° ± 0.1°C, and the effect did not vary across island size classes. This increase was strongest in summer (i.e., by 1.7° ± 0.2°C). Shrub removal increased summer soil moisture content by 6% ± 1% (dry mass basis), and moss removal increased summer soil moisture content by 2% ± 1%; this increase did not differ across island size classes (Appendix S1: Table S6).

## Discussion

By exploring forest floor CO_2_ exchange and C stocks in a long‐term plant functional group removal experiment across contrasting ecosystems, we found that the effects of shrub and moss loss were largely independent of environmental context. As such, shrub and moss removal increased net CO_2_ loss and decreased belowground C stocks consistently across a boreal forest productivity gradient. Here we explore the mechanisms and implications of these findings.

### Forest floor CO_2_ flux

Long‐term shrub removal strongly decreased gross primary productivity (GPP) across all island sizes, in agreement with our first hypothesis, and in line with previous studies showing that shrubs are major contributors to boreal forest floor GPP (Kulmala et al. 2011). Conversely, shrub removal had no overall main effect across all island size classes on ecosystem respiration (ER), contrary to our expectation that reducing plant and root‐associated respiration, and litter inputs, would diminish ER. Although plant and rhizosphere respiration can contribute substantially to forest floor respiration in boreal forests (e.g., 14%, Bergeron et al. 2009; 42%, Pumpanen et al. 2015), our findings suggest that shrub respiration was a minor part of ER, and that ER was likely instead dominated by heterotrophic and tree root respiration. Alternatively, it is possible that the loss of shrub roots in shrub removal plots could have promoted tree root biomass and thus tree root respiration, which may have partially compensated for the loss of shrub respiration. Across all islands sizes, the negative effect of shrub removals on GPP led to increased net ecosystem exchange (NEE) and thereby weakened the forest floor net CO_2_ sink. Meanwhile, moss removal had a negligible effect on average GPP, which is contrary to our expectation because mosses have been shown to contribute substantially to annual forest floor GPP (e.g., 40%; Kolari et al. 2006). However, in comparison to shrubs, the relative contribution of mosses to GPP is lowest in summer (Street et al. 2012) in part because the photosynthetic rates of mosses are lower (Kulmala et al. 2011, Bansal et al. 2012), reach their maximum at lower irradiance levels (Kolari et al. 2006), and are more dependent on ambient moisture content than those of shrubs. As irradiance and evaporation levels are highest in summer, our sampling may have been unrepresentative of the annual contribution of mosses to GPP. Similarly, we found that moss removal had no effect on average ER. However, small nonsignificant trends of moss removal increasing ER and decreasing GPP collectively led to a significantly increased NEE, i.e., moss removal added to the effect of shrub removal on weakening the forest floor net CO_2_ sink.

Although there was no main effect of shrub removals impacting on ER, there was an interactive effect of removal and island size on ER, because shrub removal substantially increased ER on medium islands, but had no effect on large and small islands. This finding does not support our hypothesis that shrub removal would decrease ER due to limiting autotrophic respiration and litter inputs, but partially agrees with our prediction that shrub removal effects would be greatest on large and medium islands where resource‐acquisitive shrub communities dominate (Wardle and Zackrisson 2005). The understory vegetation on medium islands is heavily dominated by *Vaccinium vitis‐idaea*, which produces recalcitrant litter and associated recalcitrant fungal necromass from pigmented ericoid mycorrhizal hyphae (Wardle et al. 2003, Clemmensen et al. 2015). As breakdown products from recalcitrant litter and necromass can impede microbial decomposition by forming complexes with amino acids and enzymes (Hättenschwiler and Vitousek 2000), loss of recalcitrant inputs on medium islands following shrub removal may have stimulated soil respiration. Moreover, shrub removal can increase bacterial biomass (Chen et al. 2016), which, in combination with the higher soil mineral nutrient concentration in medium islands, may have stimulated bacterial communities more in medium islands (Fanin et al. 2019), thereby also increasing heterotrophic respiration. Another possibility is that shrub removal promoted tree root biomass, and that this had a stronger effect increasing respiration in medium islands due to the high abundance of *Betula pubescens*, a species with high root respiration rates (Burton et al. 2002), on these islands.

Conversely, we found that the effect of shrub removal on reducing GPP did not vary across island size classes and was not context‐dependent. While there are large differences in maximum photosynthetic rates between dominant shrubs in the boreal forest (e.g., *V. myrtillus* rates are about four times higher than *V. vitis‐idaea*; Kolari et al. 2006, Kulmala et al. 2011), our results show that contrasting shrub communities with differing photosynthetic capacities had similar GPP, in line with Wardle et al. (2012). Our findings also contrast with studies from tundra showing a strong coupling between leaf area index and GPP (Shaver et al. 2007, Street et al. 2007), as previous work has indicated that leaf area index of the shrub community increases with island size (Wardle et al. 2003, Kumordzi et al. 2015). This suggests that physiological controls on photosynthesis were secondary to environmental controls (e.g., temperature, drought, or frost; Kulmala et al. 2011), which may have acted to constrain GPP of different shrub communities to similar levels. Further, NEE responses to shrub removal were overall consistent across the forest productivity gradient. Moreover, the small effect of moss removal on increasing NEE was not context‐dependent, likely because differences in moss biomass among the island size classes are not large.

The combined removal of shrubs and mosses had a small positive effect on annual soil temperature, but it consistently increased the daily temperature range, especially during the peak growing season, when respiration rates are highest. A more variable daily temperature range can diminish microbial respiration rates (Uvarov et al. 2006) by promoting less specialized microbial communities (Zhu and Cheng 2011), although opposite trends have also been reported (Gornall et al. 2007). Further, the combined removal of shrubs and mosses increased summer soil moisture content, which could have promoted soil respiration. Although our soil moisture data is limited (two summer sampling periods over a single year), it is consistent with previous research in the region showing drier soil under bryophytes than on bare soil, presumably due to precipitation interception and evaporation by mosses (Soudzilovskaia et al. 2011). The effect of removals on soil microclimate did not vary among island size classes, in agreement with the invariant response of GPP and NEE to removals across the gradient, but not in alignment with the observed context‐dependency of effects of shrub removal on ER. This means that the response of ER to the interactive effect of shrub removal and island size were to some extent independent of removal‐induced changes in soil surface microclimate.

### Aboveground and belowground C stocks

Moss C stocks were decreased by long‐term shrub removal, indicating that shrubs promoted moss biomass. Given that moss function is highly sensitive to desiccation, shrub presence likely facilitated moss growth by reducing ground evaporation losses through shading and reduced wind speed (Ingerpuu et al. 2005, Gundale et al. 2010). Thus, shrub removal reduced aboveground C storage of both shrubs and mosses. Further, shrub removal decreased belowground C stocks. As the observed removal‐induced increase in forest floor NEE was driven by a decrease in GPP, rather than by higher ER, our results suggest that the depletion of C stocks was due to loss of shrub litter inputs, in agreement with our second hypothesis. Carbon stocks were decreased by shrub removal on average by 0.8 kg C/m^2^ over the 22‐yr duration of the experiment. However, we note that more than one time point would be required to provide stronger evidence of a constant rate. Nevertheless, given that rates of long‐term soil C accumulation in the system are approximately eight times lower (0.0045 kg C·m^−2^·yr^−1^; Wardle et al. 2012), our results are best explained by a net loss of belowground C following shrub removal, rather than by reduced rates of C accumulation. Conversely, moss removal had no significant effect on belowground C stocks, despite moss removal increasing forest floor NEE by ~20%. This is contrary to our expectation that loss of moss litter inputs would decrease boreal forest C stores, as moss productivity accounts for 18% of understory productivity in the study system (Wardle et al. 2012), and moss litter decomposes very slowly (Lang et al. 2009). Although not statistically significant, the effect size of moss removal on reducing belowground C content (−0.3 kg C/m^2^) was substantial, and so it is possible that a longer time frame than the 22 yr duration of our experiment might be needed to accurately quantify the effect of moss removal on soil C loss. Nevertheless, our data indicates that shrub removal was the main driver of belowground C loss.

The context dependency of shrub removal on aboveground C stocks was apparent through the greater effect of shrub removal on lowering moss biomass in small islands compared to large islands (Fig. 3e–g). Lower tree cover in small islands (Wardle et al. 2003) could have led to higher forest floor exposure and to increased moss desiccation, as supported by the lower moss moisture content in small islands (189%) compared to medium and large islands (328%; Appendix S1: Table S2). Belowground C loss in shrub removal plots was similar across all islands, contrary to our hypothesis that shrub removal‐induced C loss would be greatest in large islands because of greater litter input losses where more productive shrubs dominated, and faster belowground respiration (Wardle et al. 2003, Clemmensen et al. 2015). This finding is consistent with our forest floor CO_2_ flux results showing that NEE responses to shrub and moss removal did not depend on environmental context. Further, we found that the combined removal of shrubs and mosses induced a smaller decrease in belowground C stocks than the sum of the individual effects of shrub and moss removal. This is likely due to facilitation of mosses by shrubs, whereby shrub removal not only reduced belowground litter inputs from shrubs but also from mosses, meaning that the effect of shrub removal on reducing belowground C stocks was increased by incorporating some of the effect of moss removal.

## Conclusions

We demonstrated that understory forest vegetation, and notably the shrub component, is a key contributor to net C uptake and that loss of understory plant functional groups reduces long‐term belowground C accumulation in boreal forests. Moreover, we found that the effect of shrub loss on reducing belowground C stocks is relatively consistent across a strong environmental gradient of plant productivity and soil fertility. Although understory vegetation has often been overlooked in studies on forest ecosystem processes (Nilsson and Wardle 2005), our findings provide evidence that changes in the community composition of understory vegetation that may result from global change are likely to greatly impact on the capacity of forest ecosystems to store C. Specifically, loss of shrub cover, for example due to greater susceptibility to frost resulting from reduced snow cover (Kreyling et al. 2012) or to forest management strategies seeking greater tree density (Hedwall et al. 2013), may substantially reduce the capacity of boreal forest soils to store C. Given the vast amounts of belowground C stored in boreal forests, such C loss would have important implications for climate regulation (Gauthier et al. 2015). Our results also suggest that biodiversity loss from low‐diversity ecosystems where loss of biomass is not compensated for by the remaining plant community (Kardol et al. 2018) may be particularly vulnerable to belowground C loss due to reduced litter inputs. More generally, our study addresses the current lack of understanding of how environmental context mediates the effect of plants on ecosystem processes, through showing that loss of plant functional groups has consistent effects across strong variations in plant community‐level productivity traits and soil fertility. There is a need for future work aimed at investigating the effects of losses of plant diversity on C dynamics and storage, and to compare these effects across wide ranges of ecosystems and environmental conditions, to help us predict more broadly how losses of species and functional groups will impact on the ecosystem C cycle.

## Supporting information

Appendix S1

## Data Availability

Data are available from the Dryad Digital Repository: https://doi.org/10.5061/dryad.rn8pk0p73.
