# Supplementary material for: Effects of plant functional group removal on CO2 fluxes and belowground C stocks across contrasting ecosystems
Source: Ecology. 2020 Oct 6;101(12):e03170. doi: 10.1002/ecy.3170 (PMC7757239; doi:10.1002/ecy.3170)
Supplement: Supplementary file 1 — Appendix S1 [file ECY-101-e03170-s001.pdf]

Supporting Information. Grau-Andrés, R., D.A. Wardle, M.J. Gundale, C.N. Foster, and P. Kardol. 2020. Effects of plant functional group removal on CO_2 fluxes and belowground C stocks across contrasting ecosystems. Ecology.

Appendix S1

Table S1. Selected ecosystem properties (mean values ± SE) measured at the whole island scale across the island size gradient. Within each row, numbers followed by the same letter are not statistically significant at $\alpha = 0.05$ (Tukey's test following one-way ANOVA). Data from Wardle et al. (1997), Wardle et al. (2003), Wardle et al. (2004), Wardle and Zackrisson (2005), Lagerström et al. (2009), Gundale et al. (2011), and Wardle et al. (2012).

Ecosystem property	Island size		
	Small	Medium	Large
Time since last fire (years)	3250 ± 439 a	2180 ± 385 b	585 ± 233 c
Net primary productivity (g m^{-2} yr^{-1})	159 ± 18 b	247 ± 12 a	256 ± 14 a
Tree biomass (g m^{-2})	3202 ± 462 b	7945 ± 881 a	9011 ± 488 a
Dominant tree species	*Pinus sylvestris*	*Betula pubescens*	*Picea abies*
Dominant shrub species	*Vaccinium myrtillus*	*V. vitis-idaea*	*Empetrum hermaphroditum*
Vascular plant species richness[a]	10.6 ± 0.6 a	8.6 ± 0.4 b	6.6 ± 0.6 c
Soil organic C to N ratio	32.9 ± 0.79 b	36.0 ± 1.17 ab	40.4 ± 1.18 a
Soil organic C to P ratio	759 ± 30 a	687 ± 36 ab	623 ± 20 b
Soil organic N to P ratio	23.3 ± 1.1 a	19.1 ± 0.9 b	15.4 ± 0.5 c
N release from decomposing litter (mg N g^{-1} yr^{-1})	5.8 ± 0.1 a	5.3 ± 0.1 b	5.1 ± 0.1 b
Mineral N (MIN) (μg N g^{-1})	25.3 ± 8.0 b	58.1 ± 9.2 a	38.2 ± 14.4 ab
Dissolved organic N (DON) (μg N g^{-1})	40.3 ± 4.6 b	50.7 ± 5.5 a	39.1 ± 7.2 b
MIN/(MIN+DON)	0.39 ± 0.03 b	0.53 ± 0.05 a	0.49 ± 0.04 a
Mineral P (μg P g^{-1})	24.4 ± 2.3 b	37.7 ± 4.3 a	43.6 ± 4.9 a
Membrane-extractable P (mmol kg^{-1})	4.9 ± 0.3 b	6.5 ± 0.4 a	5.9 ± 0.7 ab
Light transmission (%)	68.6 ± 2.6 a	47.1 ± 3.7 b	55.8 ± 4.5 ab

[a]Vascular plant species richness is the number of species in a 10 m radius circular plot.

Table S2. Mean (SE) of environmental variables during CO_2 flux measurement, for each island size class and plant functional group removal treatment (C, control; -M, mosses removed; -S, shrubs removed; -S-M, shrubs and mosses removed). PAR is the plot- and island size- averaged maximum photosynthetic active radiation measured during flux measurements, and MC is moisture content in dry weight basis. N = 10 for each value.

Island size	Removal	PAR max (μmol m^{-2} s^{-1})	Air *T* (°C)	Moss MC (%)	Soil *T* (°C)	Soil MC (%)
Large	C	413 (119)	21.4 (1.5)	324 (78)	11.0 (0.6)	165 (4)
	-M	517 (107)	21.4 (1.5)		11.5 (0.8)	166 (3)
	-S	352 (90)	21.4 (1.6)	262 (50)	10.8 (0.7)	173 (2)
	-S-M	358 (94)	21.4 (1.5)		11.2 (0.8)	175 (3)
Medium	C	540 (107)	22.4 (1.9)	394 (84)	13.1 (0.7)	163 (3)
	-M	473 (113)	22.3 (1.9)		13.7 (0.9)	167 (3)
	-S	408 (100)	22.5 (2.0)	339 (87)	13.0 (0.8)	166 (3)
	-S-M	387 (106)	22.5 (2.0)		13.2 (0.8)	168 (4)
Small	C	694 (117)	22.8 (2.0)	189 (24)	13.8 (0.6)	169 (4)
	-M	748 (146)	22.8 (2.0)		13.9 (0.7)	170 (3)
	-S	538 (92)	22.5 (2.0)	190 (38)	13.8 (0.6)	175 (3)
	-S-M	636 (115)	22.5 (2.0)		14.0 (0.7)	178 (4)

Table S3. Results of linear mixed effects models testing the interaction between shrub removal, moss removal and island size class on net ecosystem exchange (NEE), ecosystem respiration (ER) and gross primary productivity (GPP). Significant effects at α = 0.05 are bolded. R^2 marginal and R^2 conditional were 0.69 and 0.74 (NEE), 0.42 and 0.60 (ER), and 0.43 and 0.43 (GPP), respectively. numDF and denDF are numerator and denominator degrees of freedom.

			NEE		ER		GPP	
Term	numDF	denDF	*F*	*P*	*F*	*P*	*F*	*P*
Shrub removed (-S)	1	81	**120.6**	**<0.001**	0.0	0.87	**94.7**	**<0.001**
Moss removed (-M)	1	81	**5.2**	**0.03**	0.0	0.96	1.0	0.33
Island size (IS)	2	27	2.5	0.10	0.8	0.47	1.8	0.18
-S × -M	1	81	0.6	0.44	3.6	0.06	0.1	0.70
-S × IS	2	81	2.4	0.09	**3.3**	**0.04**	0.2	0.78
-M × IS	2	81	0.7	0.50	1.1	0.34	1.2	0.30
-S × -M × IS	2	81	0.8	0.47	1.2	0.31	0.5	0.61

Table S4. Results of linear mixed effects models testing the interaction between plant functional group removal and island size class on carbon stocks in shrubs, mosses, and in the organic soil horizon. Significant effects at $\alpha = 0.05$ are bolded. R^2 marginal and R^2 conditional were 0.05 and 0.36 (shrub), 0.24 and 0.57 (moss), and 0.63 and 0.99 (soil). numDF and denDF are numerator and denominator degrees of freedom.

C stocks	Term	numDF	denDF	*F*	*P*
Shrub	Moss removed (-M)	1	27	0.3	0.60
	Island size (IS)	2	27	**3.7**	**0.04**
	-M × IS	2	27	1.2	0.33
Moss	Shrub removed (-S)	1	27	**12.1**	**0.002**
	IS	2	27	2.2	0.13
	-S × IS	2	27	**4.8**	**0.02**
Soil	-S	1	81	**16.3**	$<$**0.001**
	-M	1	81	2.5	0.12
	IS	2	27	**24.7**	$<$**0.001**
	-S × -M	1	81	**5.8**	**0.02**
	-S × IS	2	81	0.1	0.91
	-M × IS	2	81	1.6	0.22
	-S × -M × IS	2	81	0.4	0.66

Table S5. Results of linear mixed effects models testing the effect of the interaction between island size class and season on the change in mean daily soil temperature and range due to the combined removal of shrubs and mosses. Bold values indicate significant effects ($\alpha = 0.05$). R^2 marginal and R^2 conditional values were 0.21 and 0.31 (mean daily temperature), and 0.47 and 0.72 (daily temperature range), respectively. numDF and denDF are numerator and denominator degrees of freedom.

			Mean *T*		*T* range	
Term	numDF	denDF	*F*	*P*	*F*	*P*
Season	3	72	**9.0**	**<0.001**	**54.4**	**<0.001**
Island size	2	24	0.0	0.99	1.4	0.27
Season × Island size	6	72	0.9	0.52	0.6	0.69

Table S6. Mean (SE) of soil moisture content (in %, dry weight basis) in July and August 2018. Within each row, different letters indicate significant differences at $\alpha = 0.05$, see model results[1]. N = 10 for each value.

Island size	Control	Moss removal	Shrub removal	Shrub and Moss removal
Large	160.1 (2.5) a	160.5 (1.7) a	167.2 (1.4) ab	168.9 (2.2) b
Medium	157.9 (2.6) a	163.5 (2.6) a	163.7 (2.7) a	162.7 (3.3) a
Small	162.8 (3.1) a	165.6 (2.8) a	169.0 (2.3) ab	173.3 (2.9) b

[1]Shrub removal (-S) $F_{1,81} = 32$, $P < 0.001$; Moss removal (-M) $F_{1,81} = 5.1$, $P = 0.03$; Island size (IS) $F_{2,27} = 2.0$, $P = 0.16$; -S×-M $F_{1,81} = 0.4$, $P = 0.5$; -S×IS $F_{2,81} = 2.6$, $P = 0.08$; -M×IS $F_{2,81} = 0.5$, $P = 0.6$; -S×-M×IS $F_{2,81} = 1.8$, $P = 0.18$

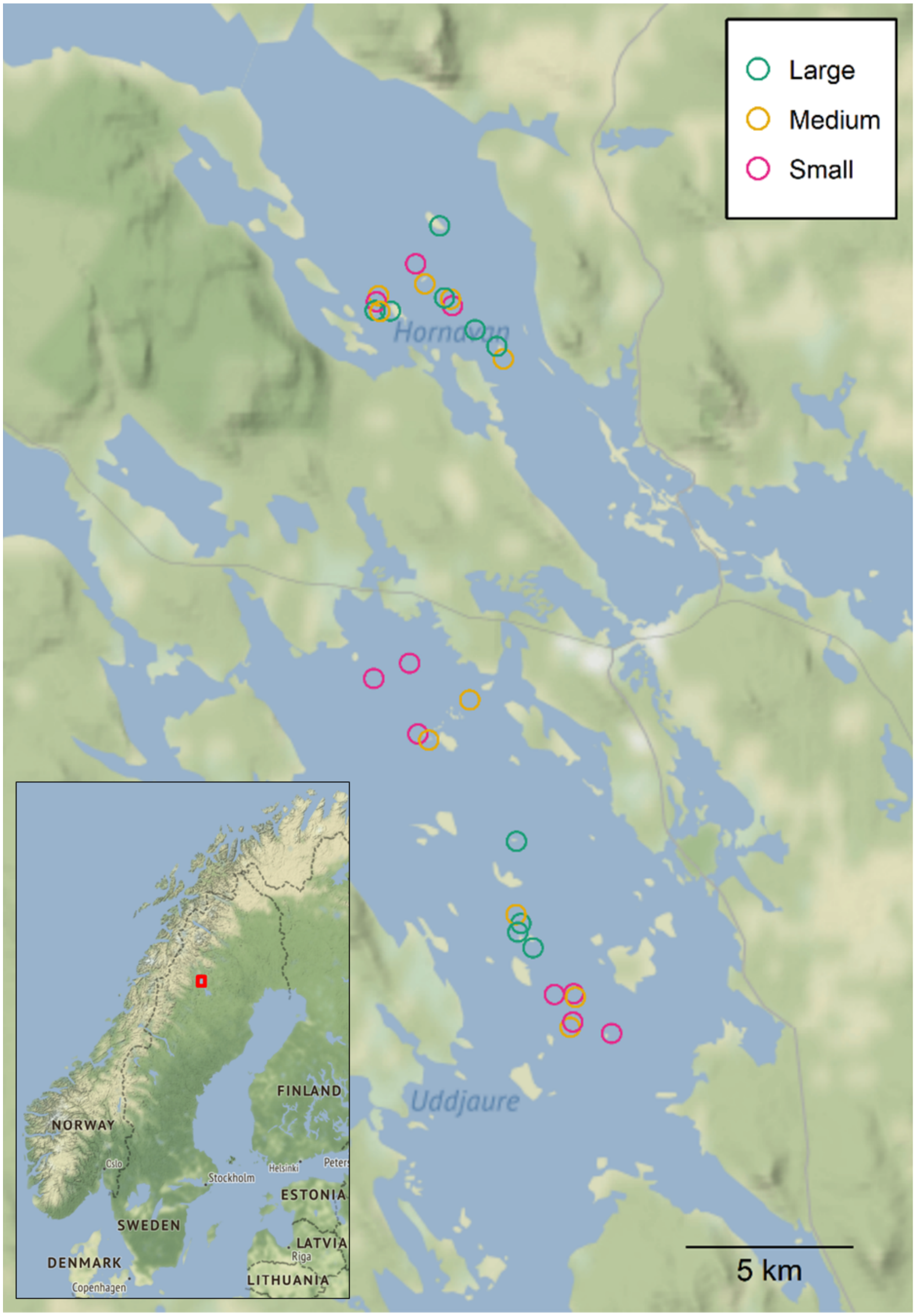

Figure S1. Location of the study site (small map) and of individual islands (big map). Colors indicate different island size classes. Map source: Stamen and Open Street Map, using the R package *OpenStreetMap* (Fellows 2019).

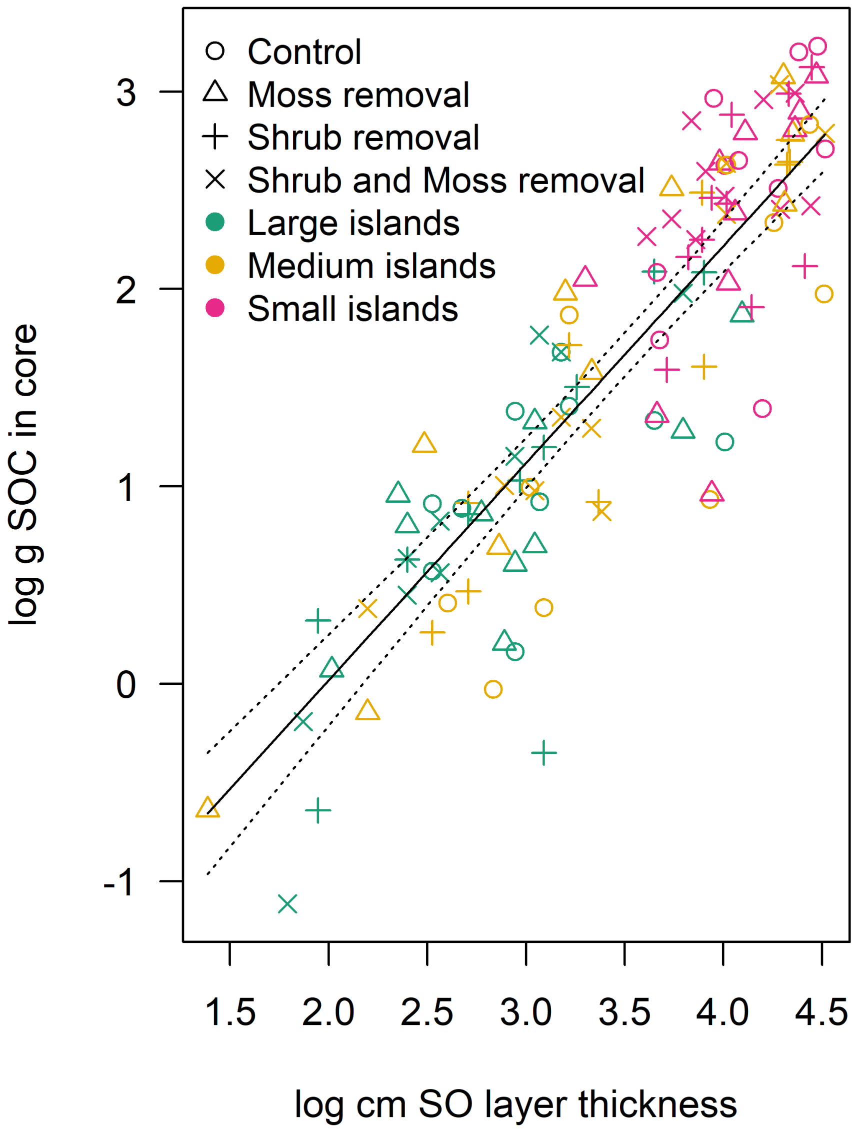

Figure S2. Soil organic (SO) carbon measured in the cores for each plot compared with the thickness of the SO horizon as measured in the hole left after the core was taken. The solid line is the predicted slope (95% CI in dotted lines) from a linear mixed effects model with log core C as a response variable and log SO horizon thickness and island size class as fixed effects, and island identity as a random effect. R^2 marginal = 0.76, R^2 conditional = 0.83.

References

Fellows, I. (2019). OpenStreetMap: Access to Open Street Map Raster Images. R package version 0.3.4.

Gundale, M. J., A. Fajardo, R. W. Lucas, M.-C. Nilsson, and D. A. Wardle (2011). Resource heterogeneity does not explain the diversity–productivity relationship across a boreal island fertility gradient. Ecography 34:887–896.

Lagerström, A., C. Esberg, D. A. Wardle, and R. Giesler (2009). Soil phosphorus and microbial response to a long-term wildfire chronosequence in northern Sweden. Biogeochemistry 95:199–213.

Wardle, D. A., G. Hörnberg, O. Zackrisson, M. Kalela-Brundin, and D. A. Coomes (2003). Long-term effects of wildfire on ecosystem properties across an island area gradient. Science 300:972–975.

Wardle, D. A., M. Jonsson, S. Bansal, R. D. Bardgett, M. J. Gundale, and D. B. Metcalfe (2012). Linking vegetation change, carbon sequestration and biodiversity: insights from island ecosystems in a long-term natural experiment. Journal of Ecology 100:16–30.

Wardle, D. A., L. R. Walker, and R. D. Bardgett (2004). Ecosystem properties and forest decline in contrasting long-term chronosequences. Science 305:509–513.

Wardle, D. A. and O. Zackrisson (2005). Effects of species and functional group loss on island ecosystem properties. Nature 435:806–810.

Wardle, D. A., O. Zackrisson, G. Hörnberg, and C. Gallet (1997). The influence of island area on ecosystem properties. Science 277:1296–1299.
